# Towards Core Competencies for Health Policy and Systems Research (HPSR) Training: Results From a Global Mapping and Consensus-Building Process

**DOI:** 10.34172/ijhpm.2020.258

**Published:** 2020-12-30

**Authors:** Meike J. Schleiff, Avanti Rangnekar, Francisco Oviedo Gomez, Gina Teddy, David H. Peters, Dina Balabanova

**Affiliations:** ^1^Department of International Health, Johns Hopkins Bloomberg School of Public Health, Baltimore, MD, USA.; ^2^University of Tennessee, Knoxville, TN, USA.; ^3^Ministry of Health, San José, Costa Rica.; ^4^School of Public Health, University of Costa Rica, San José, Costa Rica.; ^5^Center for Health Systems and Policy Research at GIMPA, Accra, Ghana.; ^6^London School of Hygiene and Tropical Medicine, London, UK.

**Keywords:** Health Systems Research, Competency Based Education, Workforce, Stakeholder Engagement

## Abstract

**Background:** As the field of health policy and systems research (HPSR) continues to grow, there is a recognition of the need for training in HPSR. This aspiration has translated into a multitude of teaching programmes of variable scope and quality, reflecting a lack of consensus on the skills and practices required for rigorous HPSR. The purpose of this paper is to identify an agreed set of core competencies for HPSR researchers, building on the previous work by the Health Systems Global (HSG) Thematic Working Group on Teaching & Learning.

**Methods:** Our methods involved an iterative approach of four phases including a literature review, key informant interviews and group discussions with HPSR educators, and webinars with pre-post surveys capturing views among the global HPSR community. The phased discussions and consensus-building contributed to the evolution of the HPSR competency domains and competencies framework.

**Results:** Emerging domains included understanding health systems complexity, assessing policies and programs, appraising data and evidence, ethical reasoning and practice, leading and mentoring, building partnerships, and translating and utilizing knowledge and HPSR evidence. The development of competencies and their application were often seen as a continuous process spanning evidence generation, partnering, communicating and helping to identify new critical health systems questions.

**Conclusion:** The HPSR competency set can be seen as a useful reference point in the teaching and practice of high-quality HPSR and can be adapted based on national priorities, the particularities of local contexts, and the needs of stakeholders (HPSR researchers and educators), as well as practitioners and policy-makers. Further research is needed in using the core competency set to design national training programmes, develop locally relevant benchmarks and assessment methods, and evaluate their use in different settings.

## Background

**Key Messages**

** Implications for policy makers**

- The field of health policy and systems research (HPSR) has rapidly expanded and diversified, creating an urgency to build training capacity to understand and address health systems challenges.
- A consultative process with HPSR educators around the world yielded a set of core HPSR competencies, but also highlighted ongoing debates.
- We found that competencies for HSPR include technical skills, abilities to address the complexities of HPSR such as those concerning collaborations and ethics, and also an emphasis on the processes for engaging stakeholders, facilitating the uptake of research findings, and a range of leadership capabilities.
- The proposed HPSR competencies should be used in a flexible manner — adapted to each context and considerate of the needs of key constituencies and institutional teaching practices.
- Having a set of recognized competencies is viewed as vital for accelerating the development of HPSR training and application to generating evidence to strengthen health systems.

** Implications for the public**

This paper aims to provide guidance for training to support a growing and evolving field of study: Health Policy and Systems Research (HPSR). This field aims to provide evidence to make health systems optimally deliver on their mandate of improving the health of communities and populations. The public can gain an understanding of the kinds of research competencies that address problems in various health systems and be better able to be involved in health systems research and advocate for training programs for health professionals who need to be able to engage with the public.

Health systems everywhere are facing unprecedented challenges as they seek to respond to ageing populations, changing patterns of disease, new technologies and models of care, and catastrophic events such as the coronavirus disease 2019 (COVID-19) pandemic. Health inequities are also persisting. More concerted efforts and collaborative research are needed in order to strengthen health systems and achieve the Sustainable Development Goals (SDGs).^1-3^ Health Policy and Systems Research (HPSR) has emerged as a scientific field that seeks to generate and apply evidence to support efforts to strengthen health systems.^4^ HPSR is defined as “… a field that seeks to understand and improve how societies organize themselves in achieving collective health goals, and how different actors interact in the policy and implementation processes to contribute to policy outcomes.”^4,5^ HPSR aims to answer questions that inform policy and practice concerns and generate lessons for application in solving health systems challenges; the field draws on many established disciplines such as medicine, economics, anthropology, sociology, and public health and utilizes a range of methods to respond to the research questions identified.^4,6^ In recognition of the pressing demands on health systems, the field of HPSR has seen rapid expansion and evolution in recent years with the creation of HPSR courses and teaching programmes worldwide as well as communities of practice. Investments in HPSR training, particularly in low- and middle- income countries (LMICs), by governments and local development agencies has also contributed to this expansion.

 Whereas significant gains have been made in HPSR methods and training, there remains large variability in what HPSR researchers do and how they should be trained. Health Systems Global (HSG), a membership organization of researchers and practitioners that seeks to strengthen HPSR, recently conducted an analysis of HSG conference participation and found progress in the breadth of content and geographical equity of participation.^7^ However, other analyses demonstrated that extensive gaps remain.^8^ Importantly, the 55% of the HSG members who responded to the survey were involved in HPSR education activities at the time, bringing to attention the need to strengthen HPSR teaching and training of individual researchers and their organizations.

 A 2014 mapping study of global HPSR teaching capacity demonstrated that training opportunities, though numerous, were concentrated in few geographic regions and the scope and modalities of HPSR training and additional assessment show similar irregularities.^9^ This and other studies demonstrated a lack of consistency within the field of HPSR teaching.^9,10^ The Collaboration for Health Policy and Systems Analysis in Africa’s (CHEPSAA’s) work^12^ and HSG’s repository of teaching and learning resources (https://courses.healthsystemsglobal.org), which includes CHEPSAA, KEYSTONE, and other pioneering HPSR training offerings, demonstrated there is still limited agreement on what the unique characteristics of HPSR are, and about what distinguishes this field from other broader areas such as global health, health services research or public health.^11,12^ What is evident is the huge variability as well as richness of HPSR training that currently exists. When also accounting for HPSR programmes in high-income settings, a lack of coherence across the field becomes apparent.^9,13^

 One route to respond to this capacity gap is to develop a competency framework, or a set of competencies which are often organized under “domains” or categories, for HSPR in order to support the development of HPSR training programmes on a wider scale. A competency is the ability to apply a set of related knowledge, skills, and abilities needed to successfully perform core work functions or tasks.^14^ Building on the advances in competency-based education domains for global public health,^14-16^ a package of core HPSR competencies can provide a point of reference for establishing high-quality education and training standards. Competencies provide comparability of desired outcomes and articulation of capabilities that researchers who complete training programmes should have across contexts. They can also provide the basis for critical review of curricula and be adapted for specific target audiences. Competency frameworks can be used as a resource and pragmatic starting point to accelerate the development of country-specific HPSR teaching offerings, such as where there are demands for scaling up national training programmes.

 This paper seeks to address these issues by providing guidance on the core elements of what HPSR researchers should be able to do. The competencies are aimed at researchers who lead, conduct and communicate HPSR research. As HPSR is usually taught at later stages of graduate or postgraduate training, a working level of research competency is assumed. We seek to make recommendations for a set of HPSR competencies which can underpin country- or institution-specific HPSR training programmes and be tailored to specific contexts and target audiences.

## Methods

 We undertook a stepwise approach, with data collection and analysis occurring in four phases, resulting in a set of interim products (see Figure). Each phase focused on a different kind of input and revision of the competency framework. First, we explored previously established HPSR-relevant competency frameworks that have been used in different contexts, and then proceeded with a series of phased engagements with an increasingly broad audience to build consensus and validate findings among the Teaching and Learning Thematic Working Group (TWG) within HSG as well as HSG membership and additional HSPR networks. The TWG (https://healthsystemsglobal.org/thematic-groups/teaching-and-learning/) is a group of global researchers and experts with experience and interest in teaching and learning for HPSR, which convenes periodic activities, contributes to HSG’s bi-annual conference, and maintains a listserv and repository of teaching and learning resources for HPSR. Our approach was iterative, enabling dialogue — not only on the focus and structure of the competency framework, but also on how it will be applied in practice to ensure buy-in from key stakeholders. We adapted our approach based on the experiences and results of each phase in order to achieve these goals.

**Figure:**
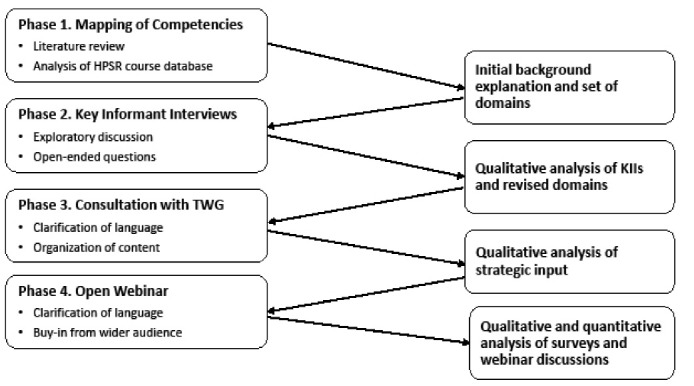
Summary of the Iterative Review and Analytical Processes Utilized in This Study. Abbreviations: TWG, Thematic Working Group; KIIs, key informant interviews.

###  Phase 1: Scoping of HPSR Competency Frameworks 

 We conducted a scoping review of existing competency sets in the fields of global health and public health, which have relevance to HPSR due to the overlap of these fields with HPSR.^15,16^ Given our target audience, we looked in particular at existing frameworks geared towards graduate and post-graduate audiences that included a component of research and/or analysis.^15-18^ A preliminary HPSR competency domain set was developed through thematic analysis of existing competency frameworks, identification of the convergence and divergence between them as well as knowledge of the field of HPSR training. For the latter, we used the HPSR training database^12^ and mapping of HPSR courses and institutions^9^ as a sampling frame to select all HPSR courses that utilized competency-based teaching or assessment. Our mapping study had previously identified that 11% (n = 18) of HPSR courses and trainings included competencies while 76% of institutions (n = 131) indicated that competencies existed at the programme level but were often not specific to HPSR.^14^ An initial draft set of global HPSR competency domains was developed to serve as the basis for discussion and input during the following stage. In addition, we employed snowball sampling to identify courses or other training programs at well-recognized centres of expertise in HPSR to identify competencies through posted information as a part of journal and working papers, websites and repositories. These included major HPSR programmes, in particular CHEPSAA, which had previously developed competencies for its own HPSR training programmes.^10,19^

###  Phase 2: In-Depth Interviews With Key Informants

 We sought to gain a detailed understanding of the field of HPSR and to solicit insight about integral HPSR competencies through in-depth interviews with diverse leaders who are actively involved in teaching HPSR. Thirteen in-depth interviews with key informants were conducted between August 2017 and March 2018. Informants were purposively selected after a careful search the HSG training database, our prior mapping exercises, and our professional networks to identify respondents with leading roles in designing, teaching and managing HPSR programmes. They were selected based on identifying key centres of expertise regarding competency-based education in fields related to HSPR and delivering institutionalized HPSR training programmes, and also identified by peers who rated them as highly qualified and engaged in HPSR teaching activities.^12^ The interviews had two parts: first, respondents were asked about their aspirations for HPSR training programmes including desired impacts and how to address current capacity gaps, how competencies are utilized across programmes that they are involved with, how existing competencies are being utilized in HPSR-related programmes within institutions, and on how a set of competencies for HPSR could or should be differentiated from public health or global health, given areas of overlap identified in Phase 1. During the second part of the interview, the respondents discussed the initial competency domains and provided feedback based on example frameworks as well as their experiences as educators in HPSR.

 All interviews were transcribed and reviewed. Two study team members (MS and DB) reviewed and analysed all transcripts and notes thematically and identified key topics associated with the study aims as well as illustrative quotes that best reflected the predominant respondents’ views. Divergent views were included to indicate areas where further consensus and research was required. The analysis compared the perspectives of respondents from high-income and LMIC in order to ensure that we captured any differences in perspectives based on these different contexts. Findings from these interviews, including on the issues that frame the state of the field of HPSR and the more general themes related to the field are presented in the results section. The competency domains were revised based on findings from these interviews.

###  Phase 3: Expert Consultations

 The initial competency domains and draft competencies were further revised following a series of consultations with experts and practitioners in the field of HPSR training to conduct a framework analysis of the drafted competency domains and specific competencies.^20^ This included a presentation and open discussion of preliminary findings at a session on building capacities for resilience in LMICs at the European Public Health Association conference in November 2017, which included participants from across the European region representing academic institutions and practitioners. Further consultations were held in May 2018 with HSG’s Teaching and Learning TWG’s Strategy Group including the founding co-chairs of the TWG, a representative from the World Health Organization (WHO), representatives of several leading HPSR institutions and networks, including CHEPSAA, and representatives of the HSG society leadership. This group represented different geographical regions—including LMIC and high-income settings—and diverse disciplinary training backgrounds though all had teaching experience in HSPR. The draft competency domains and the organization of the specific competencies were discussed via a conference call during which detailed notes were taken; and each participant provided substantive written feedback after the discussion. All inputs were analysed thematically, and key recurring points were systematically incorporated into a revised draft of competency domains and related competencies under each domain.

###  Phase 4: Open Webinar and Presentation at HSR Symposium

 At the final stage, we sought exposure and feedback from a broader audience of HPSR researchers and educators. In July 2018, a webinar was held for HSG members which involved a self-selected group of participants who were either teaching or leading HPSR training programmes or planning to develop training for HPSR in the future. The webinar was promoted via HSG email groups and listservs, LinkedIn, and the HSG newsletter, and experts/leaders of teaching and learning HPSR were also individually invited. The aim of this final phase was to receive feedback on the competency domains and the proposed specific competencies under each domain in order to highlight the highest priority areas.

 The webinar methods drew on modified e-Delphi techniques to establish consensus on the competency domains and initial draft of competencies. This process has a long history in developing a clinical consensus and it is increasingly applied achieve consensus in health policy development.^22^ This involves typically developing questions through qualitative research and a literature synthesis, and discussion between rounds of anonymous voting.^21,23^ The modified Delphi technique used in this study was appropriate because the consensus on some competency domains took time to develop and a significant learning process unfolded for many of the participants as they considered each other’s views. The webinar involved two rounds of survey questionnaires, one before and one after the interactive webinar. The anonymous pre-webinar survey (see Supplementary file 1 for the full survey tool) was distributed to all 59 webinar registrants using Google Forms; 19 responses were recorded. The survey was divided into two parts: (1) the significance of each domain to the field of HPSR and (2) the significance of a list of specific competencies within each domain to the field of HPSR. Participants were asked to rank each domain on a Likert scale and to provide comments under open-ended questions. The pre-webinar survey sought to provide inputs to help revise and restructure the competency set ahead of the webinar and to identify priority topics related to the competency set to be addressed during the webinar. After the discussion, some domains were merged as they were seen as overlapping and others were rephrased.^23^ The post seminar survey was shorter and included a more refined version of the competencies – this modification was required to reflect the evolving and diverging views – with many participants forming their views during the engagement process. The inclusion of online ‘live’ discussion was valuable to capture the richness and enable deeper understanding of the justification for the choice of competency domains and competencies and to understand if the consensus was genuine^24^ In the anonymous post-webinar survey, respondents were asked to: (1) consider the domains and sub-competencies as a set rather than stand-alone, (2) prioritize competencies for mid-level HPSR researchers, and (3) consider each competency as having levels that are programme- or audience-specific. Further revisions to wording and to how the competencies within each domain were prioritized and presented were made. The overall competencies framework was adapted based on the feedback from the webinar and post-webinar survey. Fourteen out of 59 participants submitted post-webinar survey responses.

## Results

 The final set of core HPSR competencies outlined in Table evolved over the course of the phased research process. In this section, we describe the key findings from each phase, and then discuss the final competency framework that we developed as a result.

**Table:** HPSR Researcher Competency Domains, Explanatory Domain Descriptions, and Suggested Competencies

	**Competency Statements**	**Explanatory Descriptions**
Domain 1	Understand health systems, their complexity, and policy process	*As a starting point, HPSR researchers should have a deep understanding of the nature of health systems (context, actors, financing, organization, other functions, including policy functions), and understand their nature as complex adaptive systems.*
	Competencies • Define health systems frameworks (theoretically or empirically derived) that can address health policy and program research questions in a comprehensive whole. • Compare relationships between different actors in health systems (eg, patients, health workers, managers, politicians etc) in the context of societal contracts, power and agency. • Analyse and evaluate complex interactions of elements and functions of health systems (functioning as subsystems), and how these affect policy processes. • Embed governance, stewardship and leadership as cross-cutting functions when designing and implementing research on health systems. • Deconstruct the policy processes that inform decision-making and relationships between multiple stakeholders in the health system.	
Domain 2	Assess health system-related policies and programs	*HPSR professionals should be able to ask policy-relevant research questions, design research using a wide variety of theories, methods, and approaches, and effectively carry out meaningful research on health-related policies, programs and interventions. *
	**Competencies** • Formulate policy-relevant research questions that capture and reflect the complexity of HPSR issues. • Design research that captures the multiple dimensions of context (social and political environment, values and history) shaping policies and programs and their influence on health systems and health outcomes • Apply relevant disciplinary or inter-disciplinary approaches (eg, from economics, policy analysis, epidemiology, implementation science, anthropology etc) while making connections to broader systems research theory and practice. • Integrate a wide range of theories, approaches (eg, systems thinking, implementation science, participatory research) and methods to develop questions of inquiry and design appropriate studies, including those addressing health systems complexities. • Assess the relevance of different sources and methods of data collection and analysis, building and managing teams able to apply an appropriate mix of research methods when answering HPSR questions, going beyond one’s own disciplinary expertise. • Assess when mixed methods are required to answer the research question and to combine and analyse data from these. • Integrate health systems research with analysis of and engagement with the underlying policy processes. • Generate evidence to address a problem while accepting the iterative nature of the health system design and implementation processes. • Analyse, evaluate and create strategies on how health system structures and policies can be: (*a*) designed, (*b*) implemented effectively and (*c*) adapted to overcome barriers and challenges (drawing on implementation research) while questioning conventions and assumptions.	
Domain 3	Critically appraise data and evidence related to health systems	*HPSR researchers should be able to organize, analyse, interpret, reflect on, communicate, and apply different kinds of data and evidence.*
	Competencies Critically appraise and use evidence to address health systems problems or support policy development in different settings. Apply principles of good quality data collection according to the scientific method and interpret how limitations in data collection affect interpretations about evidence in health systems Analyse data in transparent, reproducible, and robust ways according to scientific method. Synthesize knowledge obtained from across different disciplines (economics, epidemiology, anthropology, etc) to address real-world health systems issues.	
**Domain 4**	**Ethical reasoning and practice**	*HPSR researchers need the ability to identify and respond with integrity to ethical issues in diverse contexts involved in HPSR, and to promote the accountability for policies that affect people’s health.*
	Competencies • Identify and address ethical issues of HPSR related to the overall process of identifying questions, generating findings and applying lessons. This may include using appropriate methodologies for the respective settings, ensuring safety, reducing risks and enhancing benefits for participants, or the unintended use of research findings to justify decisions that may have adverse outcomes. • Demonstrate sensitivity and capacity to resolve common ethical issues related to undertaking and translating HPSR in different economic, political, and cultural contexts. • Critique research on health systems and their attributes from an equity perspective. • Enable communities, particularly vulnerable populations and those in disadvantaged settings, to have a voice in decisions concerning research questions and approaches.	
Domain 5	Lead and mentor	*HPSR researchers should be able to get teams to work together towards common purposes, foster collaboration and individual development of other professionals, and influence other health policy and systems stakeholders.*
	Competencies • Act as a leader in supporting health systems research teams, working with stakeholders involved in research, and in engaging in policy processes. • Integrate research within HPSR teaching and learning, promoting personal and institutional capacity development. • Demonstrate awareness of the role of personal and institutional positionality.	
Domain 6	Build partnerships and networks	*HPSR researchers should be able to build trusted partnerships within academia, government, and with stakeholders in civil society and across the health system. *
	Competencies • Understand the position of key stakeholders (Ministries of Health, policy-makers, health agencies, health professionals, community organizations, non-governmental agencies, business) in conducting and sharing health systems research. • Foster a collaborative research environment based on mutual respect throughout the research process, linking researchers and networks and stakeholders outside academic institutions, including advocates and those working on health systems across different sectors. • Co-produce HPSR with research participants, key local stakeholders and communities to support empowerment and health improvement. • Incorporate understanding of the social and political context of doing HPSR into how to work across stakeholder constituencies (policy, practice, media, civil society).	
Domain 7	Communicate, translate knowledge, and apply health systems evidence	*HPSR researchers should be able to incorporate evidence into ways to improve people’s health and strengthen health systems, use multiple methods of communication, and communicate effectively with different audiences.*
	Competencies • Work with stakeholders to distil policy and practice problems, create HPSR research designs to address them, and incorporating research into policy, program and health system change. • Deconstruct policy processes in order to address policy and practice problems and inform policy development and decision-making. • Promote uptake of findings through communicating HPSR effectively, engaging with various audiences and explaining meaning and relevance of findings. • Translate evidence into policy designs and implementation, including pragmatic approaches to research and HPSR for program monitoring and evaluation. • Promote health systems development leading to improved health status and access to essential services.	

Abbreviation: HPSR, Health Policy and Systems Research.

###  HPSR Competencies Scoping (Phase 1)

 The only model that fit our requirements of an explicit HPSR competency framework was the set of HPSR leadership capabilities and competencies by the CHEPSAA and specific to African Health Policy and Systems Research and Analysis (HPSR+A).^25,26^ It was fully developed and linked to specific elements of the training programmes and materials. The model was designed for an African context, but with wider relevance as it operationalized elements covered in the learning objectives of other HPSR courses. For these reasons it was chosen as a reference model. After canvassing a broad range of potential competency frameworks, two further relevant frameworks were identified: the global health competency model developed by the Association of Schools of Public Health and the Education Committee’s Master’s degree in Public Health core competencies.^16^ Although not specific to HPSR, both have significant overlap with HPSR-focused course, CHEPSAA as well other courses (as reflected in the learning objectives).

 The three competency frameworks were useful as a reference for developing an initial HPSR competency set primarily due to their similarity in content, and appropriateness given our research aim and target audiences. The emphasis and organization of the domains across the frameworks differed, however, and provided a useful basis for deliberation about what is needed in a framework for HSPR. In all three frameworks, the competency domains and detailed competencies under each domain were organized in a table format and one also had a visual representation. The CHEPSAA framework took a unique approach to organizing the content and presentation of the domains and sub-competencies within domains. This framework was developed to support leadership training for HPSR and therefore emphasized personal qualities including communications and listening skills, writing and teaching skills, networking, and research skills. Similarities in the audiences of the different frameworks served as an additional rationale for selecting the three samples as a basis for the development of the HPSR competencies. While Association of Schools of Public Health’s model is geared towards master’s level public health students, CHEPSAA’s framework is geared towards early career researchers including graduate students and post-graduate professionals.

###  Qualitative Interviews (Phase 2)

 The in-depth interviews provided important context on the state of the field of HPSR and several fundamental considerations to inform how the HPSR competency framework and training strategies going forward fit into the overall growth and evolution of the field of HPSR. They focused on the priorities for the HPSR competency domains in the context of broader considerations for the state of development of the field of HPSR and whether the field itself is “ready” to have a set of competencies. The interviews also discussed the role of HPSR researchers in producing findings but also communicating and engaging with different constituencies to inform policy, and the potential challenges for meaningful and consistent assessment across competencies. Below, we present key emerging themes.

####  HPSR: An Evolving Field that Requires Modern-day Skills 

 Multiple respondents perceived the field of HPSR as young and continuously growing, building on two decades of dramatic change. As such, the capacity needs of the field have evolved and will continue to do so in the years to come; thus, the competency framework needs to build from a deeper understanding of the evolution and diverse application and priorities across users and contexts. Perceptions are changing around the value and role of HPSR, though lingering critiques and traditional perspectives remain:

*“We had a group of traditional professionals—important researchers. They were old fashioned—from the reform in the 1980s. They were more political than scientific. Now, new researchers can balance these skills better. We had a tension between these groups. After some crisis, we are in the direction of a civilized consensus within the institution. It is good for students to see these different points of view—we don’t want to protect them. It is a good, more consistent direction from a scientific point of view and also considering the...reality as it is now”* (LMIC, academic).

 As the next generation of HPSR researchers is trained, emphasizing the historical trajectory, the potential for growth, and the value of the HPSR field can help guide continued evolution and growth.

 Respondents offered both larger philosophical as well as specific skill-oriented perspectives on what the particular challenges and opportunities of working in HPSR are and the associated skillsets needed. While the field draws on many different disciplines including global health and public health, there was agreement that the kind of questions that HPSR aims to address as well as the kind of impact that it can yield present unique opportunities but also challenges:

*“[HPSR] takes quite extraordinary individuals. It is not enough to have excellent epidemiologists with wonderful designs that sit at the computer and deliver great reports published in wonderful papers. There is something more with the best in HPSR, and we need to be very careful with this. It is not people like me, it is people in the new generation. The new generation, we expect to get both solid competence in mixed methods with some focusing on both sides, but you also want them to have communicative competence and leadership attributes”* (HIC, academic).

 Ultimately, as the HPSR community continues to explore what sets it apart and also how best to integrate with diverse stakeholders and respond to real-world challenges, there should be continued efforts to more clearly articulate the added value of high quality HPSR and the skills and knowledge that are required to deliver it.

####  The Role of Disciplinary Skills in a Multi-Disciplinary Field

 Ultimately HPSR is considered to involve ‘social research’ competencies. However, respondents offered different perspectives on the appropriate role for prior discipline-specific training and specific disciplinary backgrounds that may be well aligned to enable high quality HPSR. This was important as we developed the competency framework and considered what prior knowledge and skills could be assumed for graduate-level and professional learners in HPSR. While having expertise in a particular discipline can be quite valuable, being able to understand, appreciate, and listen to ideas and considerations across disciplines was generally seen as being at the core of effective HPSR:

*“There isn’t one blueprint. Everyone brings their own unique background and context with them. There is not a core HPSR training—you “arrive there” from elsewhere and bring that background with you. We cannot be prescriptive about what this prior background should look like because it is very individualized”* (LMIC, academic/international advocate).

 Respondents also provided differing perspectives on the appropriate or desirable backgrounds for HPSR, but generally saw the value in having an in-depth background in at least one discipline. Furthermore, respondents agreed on the benefits of engaging with colleagues and drawing upon different disciplines in order to most effectively ask questions and understand complex health system problems. One respondent drew a parallel between how a family physician needs to understand enough about other specialties in order to know when to refer a patient for additional tests or treatment, and whether a similar kind of thought process can be applicable to HPSR.

 Some respondents argued to avoid pitching different disciplines against each other and instead finding ways to draw on many of these in a training programme, enabling the students to grapple with real-life issues:

*“If someone is coming into the field of HPSR, they are often trying to lobby for their piece of the pie or your approach to things. If someone has never come across any economics, for example, before and it has never been part of their training, it is an enormous battle to first of all educate that person and then you’ve got to convince them…If you’re talking to someone with the background who has covered all of the technical pieces, they will say, ‘oh, yes, I can see your point.’ And then you can move to the crux of the matter*” (HIC, academic).

####  Role of Researchers in Communicating and Using Evidence From HPSR

 Respondents provided several perspectives on the roles and responsibilities of researchers in engaging with other stakeholders to ensure effective communication and use of evidence produced through HPSR. While there is a broad debate on the importance of closing the gap between generation and use of evidence, the need for the HPSR competencies to include both these aspects came out as a common theme as an essential component of HPSR. Differing perspectives came out in both the key informant interviews and also in the Phase 3 survey results. Some felt that this was a core aspect of HPSR, while others felt it was an important role for researchers in general and not unique to HPSR. There were also differing perspectives on to what degree HPSR researchers should engage in advocacy and what kinds of added value and/or biases this might result in:

*“When I was a senior advisor to the minister, frequently we had to welcome people from academia and other places who wanted to show us results from studies, and the conclusion at the end was: ‘So what?’ ‘ So what do you expect us to do with this knowledge now?’…And the most common answer we would get is: ‘I am a researcher. … I just deliver the information’” *(LMIC, public sector).

 Overall, respondents felt that it was valuable for HPSR researchers to engage in the full cycle of research, including translation into practice and engaging other relevant stakeholders. However, best practices to undertake this process at different levels and the degree to which HPSR researchers have ethical or professional obligations to be advocates and focus on knowledge translation versus generation were areas of continued debate.

####  Consensus-Building (Phases 3 and 4)

 Given the novelty and contestation of the competencies for the field of HPSR, findings from the qualitative interviews suggested a strong interest and enthusiasm for continuing dialogue around competencies. As we solicited perspectives from key stakeholders, our learning through phases 3 and 4 included (1) validation of the utility and the demand for such a general framework to guide individual countries, organizations, and leaders in developing HPSR curricula as well as (2) convergence around several ways of framing the relationships between the competency domains as well as (3) what makes training for HPSR unique. We report the findings from phases 3-4 in tandem as they built upon each other and took place somewhat concurrently.

 During our TWG’s strategy group meeting, participants provided feedback regarding definitions and differentiating between competency domains, orienting sub-competencies towards higher levels of Bloom’s Taxonomy appropriate to our target audiences, and considering the ability to assess competencies within each domain. We filled gaps identified by the strategy group and in response to questions and ideas from conference participants. For example, we added additional competencies under the domain related to assessment of health system policies and programs as well as under ethics in order to enhance the focus of the framework on considering the complexities of working with a range of stakeholders to design research and ensure that it would benefit the desired target audiences – particularly vulnerable populations. There was also a focus, particularly with participants at conference sessions, to test direct application of the competencies into contextually relevant HPSR training programmes and continue to revise the framework based on that experience.

 The webinar with HSG members was a central point for consensus building as it included a broad base of participants, including educators and programme leaders but also recipients of HPSR training. The webinar resulted in a rich discussion and generated new perspectives and insights, including the option of thinking about the competencies as an iterative cycle that learners and researchers follow from understanding health systems contexts and identifying research questions through to dissemination and uptake of research findings. Another key recommendation was about the value of structuring the competencies not as a list of stand-alone knowledge and skills areas, but in relation to each other and as a part of an inter-related package of competencies needed for effective HPSR work. Illustrative of this evolution was the pre- and post-webinar survey, where respondents did not rate all the competency domains as equally important to the field of HPSR and provided insightful comments as to the reasons for this and the changes in their views before and after the webinar. Respondents were asked about the relevance of each domain to the field of HPSR; responses were divergent as the “lead and mentor” and the “building partnerships and networks” domains were rated as less highly relevant to HPSR in the post webinar survey compared to the pre-webinar survey responses. These findings are in contrast to many of the key informants’ perspectives, which instead put embeddedness, leadership, and collaborative work at the very heart of the field of HPSR.

 The different priorities observed in engaging with multiple stakeholders during Phases 3-4 highlights that HPSR competency domains have different relevance across contexts and apply to different types of training leaders and audiences. Thus, the weights and importance of particular domains and their competencies will vary in each setting, and it is not possible to derive a single definite answer that works for all audiences and learners in HPSR. Ultimately, the competency domains identified through this study reflect a consensus on a global reference point, but they can and should be adapted to specific contexts and be flexible to audience needs.

####  HPSR Competency Domains and Core Competencies: A Final Set

 Although each competency may relate to other disciplines and fields in global health or public health, the study participants identified that the HPSR competencies related to specific knowledge, skills and work practices in the field have to be taught and applied as *a coherent set *that underpins rigorous HPSR.

 The final competency framework (Table) was considered to be a set of core HPSR competencies by HPSR training programme leaders and respondents actively involved in designing and providing HPSR offerings. The table provides explanatory descriptions of each domain as well as the set of competencies that fall under each one. An important caveat is that there are differences in what competencies mean for different disciplines and in diverse settings; HPSR training programmes need to adapt their competencies to fit these different contexts. While they will have differing relevance across contexts, they can serve as a reference point for the continued development of HPSR training initiatives. Ultimately, these domains reflect a conceptualization of the role of researcher as either being a detached observer or a leader and influencer of a rapidly developing health systems agenda.

 The order of the competencies is not prescriptive, and the domains of the framework often do not get taught or utilized sequentially in practice; for example, leadership and mentoring can be valuable at all stages of a research process, and ethical considerations may arise at any time. The exact focus of the competencies and their sequencing will vary in each context to ensure best fit and outcomes. The framework can be seen as an analytical tool that can be adapted to diverse contexts, and which should follow and inform—and not dictate—the actual starting point, sequence of needs, and priorities for a particular audience.

 Particular emphasis was given to the leadership role of HPSR researchers, which enables them to undertake a full cycle of HPSR, including the policy engagement and communication of findings towards generation of new research agendas. However, a common theme was the importance of co-production of research and working together in partnerships and networks. Finally, the communication of findings to a wide range of stakeholders is a key domain of the HPSR competencies set, which in itself leads to knowledge update and a new cycle of generation of policy questions.

 Importantly, there are a few overlaps between competencies in each domain and the methods utilized in each phase of the study elicited diverse preferences among the respondents—therefore the grouping of competencies sets under each domain should not be taken to be rigid. For example, some competencies under the Domain 1 on understanding health systems are also relevant for Domain 6 on building partnerships and networks. In addition, Domain 5 on leading and mentorship relates to the final Domain 7 and communicating and translating knowledge as both rely on foundational skills related to capacity strengthening and effectively engaging with peers, junior colleagues, and other stakeholders.

## Discussion

 This paper sought to identify and describe a competency framework for HPSR drawing on qualitative data enquiry and consensus building exercises. This framework was viewed by participants as critical to the development of HPSR training programmes worldwide.

 We acknowledge the following limitations. Given that HPSR training boundaries and priorities continue to be refined and evolved, we started from a very broad understanding of the field and of competency-based education, and purposefully aimed to capture a diversity of perspectives, including leaders in the field of HPSR globally as well as other key HPSR researchers with interest in training and learning. We sought to ensure diversity through conducting online events and providing opportunities for written feedback over periods of time, but may not have captured a full range of critical thinking on HPSR teaching and learning. We also did not capture a level of detail to specify the particular dimensions of current debates related to inequity, racism, sexism and gender inequities, and other social issues around diversity and inclusion that merit attention across educational endeavours. We also recognized that, while HPSR is a multi-disciplinary field, there was limited consideration of multi-sectoral research or the importance of specifically collaborating to answer research questions across sectors. These kinds of research questions are critical to inform the SDG agenda and we see HPSR starting to given attention to these issues.

 Each phase of this study process yielded important and different feedback, which contributed to the final version of the competency framework. One of the most challenging aspects of the study was to carefully consider all of the inputs that were often significantly divergent and to work towards agreement. As the field of HPSR continues to grow, these competencies can serve as a basis for advancing debates about what competencies are required across contexts and to inform further research about how best to strengthen capacity for working within this complex field. As we saw during each phase of this study, the need to adapt and contextualize HPSR training in order to be responsive to the priorities of each health system is a central theme that came out even as we engaged with many respondents who had different understandings and experiences of the role and meaning of competencies to guide curriculum development. Further work is needed to refine how we describe and use competencies across contexts, and to also develop levels of achievement within each competency domain, or alternative approaches to assessing capacity strengthening across these domains. As the field of HPSR continues to be constructed and refined, delving deeper into the best ways to assess competence across the diversity of people engaged in HPSR is a challenge that requires additional work and is a valuable effort to ensure that the competencies included in the framework are as relevant and measurable as possible.

 Our results demonstrated some important evolutions in thinking and priorities, as did our deliberations at each stage in the study process. At stages one and two, we noticed the limited work specific to HPSR in this space, while also recognizing CHEPSAA’s ground-breaking work on which we have aimed to build. In stage three, our consultations reinforced the demand for such a framework and enabled us to capture additional perspectives, including from teachers and public sector workers who want to enhance HPSR capacity across different contexts. In stage four, we identified that different groups of participants in the webinar and pre- and post-webinar surveys may have had different priorities, particularly regarding the role of leadership in HPSR. Research experts leading HPSR research and capacity building initiatives may be attributing more importance to the leadership of overall HPSR programmes in contrast to HSG members who may be focused on research/training in relation to specific areas of the framework.

 Enthusiasm for the framework and recommendations to adapt and use it have been strong from early on in the development process. The final competency domains and the top-ranked sub-competencies for each domain were presented and discussed at the TWG’s organized session at the Health Systems Research Symposium in Liverpool, United Kingdom in October 2018, receiving positive reception and thoughtful questions by participants. In addition, the draft framework was utilized to inform the development of a national training plan for HPSR in Malaysia supported by the WHO. The HPSR domains from November 2017 were considered during a process led by the Institute for Health Systems Research at the Ministry of Health of Malaysia in collaboration with London School of Hygiene and Tropical Medicine (LSHTM) and WHO which sought to develop a plan for a nation-wide HPSR training programme. The set of domains presented in this paper were used to develop HPSR competencies tailored to the national context of Malaysia. These domains are being used to further adapt the competencies and accelerate the development of HPSR in Malaysia and in the Western Pacific region through consultative processes with key stakeholders and communities in 2020-2021. The process will help to test the adaptability and versatility of the framework across different contexts.

 Within a young and evolving field that includes a wide diversity of disciplines and contexts where research is being implemented, it can be important to develop competencies and training content for different audiences. The competency framework is not intended as a blueprint for HSPR training, but as a resource and benchmark which can underpin and enable the development of institution- and region-specific competencies as well as competencies with different thematic foci. Thus, the findings presented in this paper are not an end point, but rather a step in an ongoing process of developing an operational competency framework for the field of HPSR. It builds on the pioneering work by CHEPSAA as well as parallel efforts in the field of global public health.^15,16,26^ Overall, the findings suggest that we will need to consider capacity strengthening activities that leverage the particular strengths of HSG and other leading organizations and networks engaged in HPSR as a global society of people working in HPSR in diverse settings around the world.

## Conclusion

 This paper argues that clearly identifying the skills and knowledge that pertain to HPSR and embedding these into a core competency set is critical to supporting HPSR capacity strengthening. While we propose a set of HPSR competencies derived through consensus building processes within key communities of practice, these are a reference point that should be adapted, implemented, and assessed to fit different country contexts. The field continues to evolve, but we believe this set of competencies can enable research leaders and educators to design and implement effective HPSR training that supports learners to produce stronger evidence, effectively engage in the entire cycle of research generation, and inform the strengthening of health systems.

## Acknowledgements

 This work was undertaken as a collaborative effort within the Thematic Working Group for Teaching and Learning Health Policy and Systems Research, HSG. We would like to thank all of the respondents who engaged in this participatory process – often multiple times during the study period. We appreciate the insightful perspectives, challenging questions, and commitment to helping achieve a product that will have value for the field. We also wish to thank the HSG secretariat and many Teaching and Learning Thematic Working Group members for their support and encouragement. Finally, we appreciate Professor Lucy Gilson’s very helpful feedback and questions on an earlier version of the manuscript. The named authors alone are responsible for the views expressed in this publication.

## Ethical issues

 London School of Hygiene and Tropical Medicine (LSHTM) ethics committee reviewed and approved this study (LSHTM ethics ref: 14356/RR/8627).

## Competing interests

 Authors declare that they have no competing interests.

## Authors’ contributions

 MJS, AR, and DB collected and analysed the data for this paper. MJS and DB wrote the first drafts of the manuscript. All co-authors had substantive inputs to drafts of the manuscript during at least two points in time. AR supported with editing and formatting of the manuscript.

## Supplementary files

Supplementary file 1. Pre- and Post-webinar Survey Questions.
