# Supplementary material for: Towards Core Competencies for Health Policy and Systems Research (HPSR) Training: Results From a Global Mapping and Consensus-Building Process
Source: Int J Health Policy Manag. 2020 Dec 30;11(7):1058–68. doi: 10.34172/ijhpm.2020.258 (PMC9808165; doi:10.34172/ijhpm.2020.258)
Supplement: Supplementary file 1 — Pre- and Post-webinar Survey Questions. [file ijhpm-11-1058-s001.pdf]

Supplementary file 1. Pre- and Post-webinar Survey Questions

The following competencies were considered to be essential HPSR competencies – either by HPSR training programmes or by respondents actively involved in designing and providing HPSR offerings. The purpose of this form is to help us identify which of these HPSR competencies are considered essential by many in the field, and which are supplemental or more general competencies and not core to HPSR.

The following questionnaire will ask that you rate how significant the listed HPSR competencies and their associated domains are to the field of HPSR:

Domain 1: Ability to conceptualize health systems as complex adaptive systems composed of interactive subsystems, influenced and embedded into policy and context, and to design and undertake research that draws on these concepts, to address policy-relevant questions.

1. How significant is Domain 1 to the field of HPSR (Highly Significant/Central to HPSR, Moderately Significant/Neutral/Indifferent, Not Significant/Not Central to HPSR)?
2. How significant to the field of HPSR are the following competencies under Domain 1 (Highly Significant/Central to HPSR, Moderately Significant/Neutral/Indifferent, Not Significant/Not Central to HPSR)?
    a. Analyze and evaluate complex interactions of elements and functions of health systems (functioning as subsystems), and how these affect policy processes.
    b. Define a comprehensive health systems model (or a conceptual framework) that can address health policy and program research questions.
    c. Embed governance, stewardship and leadership as cross-cutting health system functions when designing and implementing research.
    d. Analyze, evaluate and create strategies on how health system structures and policies can be: a) designed, b) implemented effectively and c) adapted to overcome barriers and challenges (drawing on implementation research) while questioning conventions and assumptions.

e. Deconstruct the policy processes that inform decision-making and relationships between multiple stakeholders in the health system.
f. Compare relationships between different actors in health systems (patients, health workers, managers, politicians etc.) in the context of societal contracts, power and agency.
g. Integrate a wide range of theories and approaches (systems thinking, implementation science, economics, etc.), when appropriate by identifying questions and methods that explore complexities.
h. Critique research on health systems and their attributes from an equity perspective.
i. Design research that captures the multiple dimensions of context—social and political environment, values and history—that shape health systems and health outcomes.
j. Assess the relevance of HPSR findings across contexts.

Domain 2: Ability to undertake rigorous HPSR research.

3. How significant is Domain 2 to the field of HPSR (Highly Significant/Central to HPSR, Moderately Significant/Neutral/Indifferent, Not Significant/Not Central to HPSR)?
4. How significant to the field of HPSR are the following competencies under Domain 2 (Highly Significant/Central to HPSR, Moderately Significant/Neutral/Indifferent, Not Significant/Not Central to HPSR)?
    a. Apply principles of good quality data collection according to scientific method.
    b. Analyze data in transparent, reproducible, and robust ways according to scientific method.
    c. Create health systems models or conceptual frameworks that can be applied to policy and programmatic research questions.

Domain 3: Ability to take a leadership role in HPSR, shape the field, create local, national and global research agendas, and implement HPSR in order to engage relevant stakeholders.

5. How significant is Domain 3 to the field of HPSR (Highly Significant/Central to HPSR, Moderately Significant/Neutral/Indifferent, Not Significant/Not Central to HPSR)?
6. How significant to the field of HPSR are the following competencies under Domain 3 (Highly Significant/Central to HPSR, Moderately Significant/Neutral/Indifferent, Not Significant/Not Central to HPSR)?
    a. Act as a leader at the systems level, working with governance processes.
    b. Question the status quo.
    c. Understand the position of key stakeholders (MOH, institutions, health staff, community) in conducting and sharing the research.
    d. Demonstrate awareness of the role of personal and institutional positionality.

Domain 4: Ability to develop and apply appropriate methodologies to answer policy-relevant research questions

7. How significant is Domain 4 to the field of HPSR (Highly Significant/Central to HPSR, Moderately Significant/Neutral/Indifferent, Not Significant/Not Central to HPSR)?
8. How significant to the field of HPSR are the following competencies under Domain 4
    a. Formulate policy-relevant research questions that capture and reflect the complexity of HPSR issues.
    b. Apply a disciplinary approach (e.g. in economics, policy analysis, epidemiology, implementation science, anthropology etc.) in order to make connections to broader systems research theory and practice.
    c. Assess the relevance of, and apply an appropriate (mix of) methods, when answering research questions related to health systems elements or subsystems.
    d. Ability to judge when mixed methods are required to answer the research question and to combine and analyze data from these.
    e. Integrate health systems research with analysis of the underlying policy process related to policy development, planning and implementation.
    f. Synthesize knowledge obtained from across different disciplines (economics, epidemiology, anthropology, etc.) to address real-world health systems issues.

  g. Generate evidence to address a problem while accepting the iterative nature of the health system design and implementation processes.
  h. Critically appraise and use evidence to address health systems problems or support policy development.

Domain 5: Ability to recognize and address HPSR-specific ethical issues, including considerations for research settings, and voice within the research and decision-making process.

9. How significant is Domain 5 to the field of HPSR (Highly Significant/Central to HPSR, Moderately Significant/Neutral/Indifferent, Not Significant/Not Central to HPSR)?
10. How significant to the field of HPSR are the following competencies under Domain 5 (Highly Significant/Central to HPSR, Moderately Significant/Neutral/Indifferent, Not Significant/Not Central to HPSR)?
    a. Identify and address ethical issues of HPSR (including the process of generating and applying findings).
    b. Demonstrate sensitivity and capacity to resolve common ethical issues related to undertaking and translating HPSR in different economic, political, and cultural contexts.
    c. Enable communities, particularly vulnerable populations and those in low-resource settings, to have a voice in the ethics of research questions and approaches.

Domain 6: Ability to co-produce research and cultivate skills to develop, sustain, and continually adapt partnerships within changing contexts.

11. How significant is Domain 6 to the field of HPSR (Highly Significant/Central to HPSR, Moderately Significant/Neutral/Indifferent, Not Significant/Not Central to HPSR)?
12. How significant to the field of HPSR are the following competencies under Domain 6 (Highly Significant/Central to HPSR, Moderately Significant/Neutral/Indifferent, Not Significant/Not Central to HPSR)?

a. Foster a collaborative research environment linking: a) researchers and networks in all stages of research b) stakeholders outside academic institutions, including advocates and c) those working on health systems across different sectors. Base collaboration on mutual respect.
b. Integrate research within HPSR teaching and learning through ensuring personal and institutional capacity development.
c. Co-produce HPSR with research participants, key local stakeholders and communities to support empowerment and health improvement.
d. Incorporate understanding of the social and political context of doing HPSR into how to work across constituencies (policy, practice, media, civil society).

Domain 7: Ability to communicate and enable policy translation of HPSR findings into policy processes, engaging with varied constituencies from policy makers to research participants.

13. How significant is Domain 7 to the field of HPSR (Highly Significant/Central to HPSR, Moderately Significant/Neutral/Indifferent, Not Significant/Not Central to HPSR)?
14. How significant to the field of HPSR are the following competencies under Domain 7 (Highly Significant/Central to HPSR, Moderately Significant/Neutral/Indifferent, Not Significant/Not Central to HPSR)?
    a. Work with stakeholders to distill policy and practice problems, create HPSR research designs to address them, and implement research and health system change.
    b. Deconstruct policy processes in order to address policy and practice problems and inform policy development and decision making.
    c. Promote uptake of findings through communicating HPSR effectively, engaging with various audiences and explaining meaning and relevance of findings.
    d. Translate evidence into policy, including pragmatic approaches to research and HPSR for program monitoring and evaluation beyond academic research.
    e. Promote health systems development leading to improved health status and access to essential services.
